# Characterization of membranous and cytoplasmic EGFR expression in human normal renal cortex and renal cell carcinoma

**DOI:** 10.1186/1423-0127-16-82

**Published:** 2009-09-12

**Authors:** Yeong-Shiau Pu, Chao-Yuan Huang, Yi-Zih Kuo, Wang-Yi Kang, Guang-Yaw Liu, A-Mei Huang, Hong-Jeng Yu, Ming-Kuen Lai, Shu-Pin Huang, Wen-Jeng Wu, Shean-Jaw Chiou, Tzyh-Chyuan Hour

**Affiliations:** 1 Department of Urology, National Taiwan University College of Medicine, Taipei, Taiwan, Republic of China; 2 Department of Oncology, National Taiwan University College of Medicine, Taipei, Taiwan, Republic of China; 3 Institute of Biochemistry, Kaohsiung Medical University, Kaohsiung, Taiwan, Republic of China; 4 Center of Excellent for Environmental Medicine, Kaohsiung Medical University, Kaohsiung, Taiwan, Republic of China; 5 Institute of Immunology, Chung Shan Medical University, Taichung, Taiwan, Republic of China; 6 Department of Pathology, Kuo General Hospital, Tainan, Taiwan, Republic of China; 7 Department of Urology, Kaohsiung Medical University Chung-Ho Memorial Hospital, Kaohsiung Medical University, Kaohsiung, Taiwan, Republic of China

## Abstract

Metastatic renal cell carcinoma (RCC) is highly resistant to conventional systemic treatments, including chemotherapy, radiotherapy and hormonal therapies. Previous studies have shown over-expression of EGFR is associated with high grade tumors and a worse prognosis. Recent studies suggest anticancer therapies targeting the EGFR pathway have shown promising results in clinical trials of RCC patients. Therefore, characterization of the level and localization of EGFR expression in RCC is important for target-dependent therapy. In this study, we investigated the clinical significance of cellular localization of EGFR in human normal renal cortex and RCC. RCC and adjacent normal kidney tissues of 63 patients were obtained for characterization of EGFR expression. EGFR protein expression was assessed by immunohistochemistry on a scale from 0 to 300 (percentage of positive cells × staining intensity) and Western blotting. EGFR membranous staining was significantly stronger in RCC tumors than in normal tissues (*P *< 0.001). In contrast, EGFR cytoplasmic staining was significantly higher in normal than in tumor tissues (*P *< 0.001). The levels of membranous or cytoplasmic EGFR expression in RCC tissues were not correlated with sex, tumor grade, TNM stage or overall survival (*P *> 0.05). These results showed abundant expression of membranous EGFR in RCC, and abundant expression of cytoplasmic EGFR in normal tissues. EGFR expression in RCC was mostly located in the cell membrane, whereas the EGFR expression in normal renal tissues was chiefly seen in cytoplasm. Our results suggest different locations of EGFR expression may be associated with human renal tumorigenesis.

## Introduction

Renal cell carcinoma (RCC) arises mainly from renal tubular epithelia [1]. Surgical resection of the diseased tissue has been considered the only curative treatment [2]. Metastatic RCC is highly resistant to conventional systemic treatments, including chemotherapy, radiotherapy and about 10-20% of patients respond to cytokine-based immunotherapy [3]. Development of targeted therapies in renal cell cancer is largely due to the fact that a growing understanding of the underlying molecular biology of RCC has established the vascular endothelial growth factor (VEGF) and mammalian target of rapamycin (mTOR) pathways as relevant therapeutic targets in RCC [3,4]. Despite the treatments available nearly all patients die of metastatic disease. Many studies have demonstrated genetic and environmental factors lead to RCC occurring during a protracted period of tumorigenesis [4]. It seemed desirable to identify and characterize potential molecular markers appearing during of tumorigenesis which might provide rapid and effective possibilities for early detection of RCC [5].

Epidermal growth factor receptor (EGFR) is classified into a family of four closely related cell membrane receptors: EGFR (HER1; ErbB1), HER2 (ErbB2), HER3 (ErbB3), and HER4 (ErbB4) [6]. These receptors are glycoproteins of transmembrane with an extracellular ligand binding domain and an intracellular domain with tyrosine kinase activity involved in signal transduction [7]. EGFR activation induces the cell cycle progression, inhibition of apoptosis and angiogenesis, promotion of invasion/metastasis, and other tumor promoting activities [8,9]. EGFR overexpression has been associated with an aggressive clinical course in many cancers [10-12]. RCCs frequently show EGFR immunoreactivity [13,14]. Previous studies have shown p-regulation of EGFR is one of the common events in RCC tumorigenesis [15]. Over-expression of EGFR is thought to play an important role in tumor initiation and progression of RCC, since up-regulation of EGFR has been associated with high grade and a worse prognosis [16,17]. This is particularly interesting because recently, anticancer therapies targeting the EGFR pathway have shown promising results in clinical trials of RCC patients [18,19].

Recent studies suggest the existence of a novel role of EGFR signaling pathway where activated EGFR undergoes nuclear translocalization, subsequently regulating gene expression and potentially mediating specific cellular processes [20-22]. This new role of EGFR is distinct from the well-known traditional EGFR involving transduction of mitogenic signals through activating multiple signaling cascades [23]. These results point out EGFR may play a novel role as a cytoplasmic/nuclear shuttling transcription factor in tumor progression [24]. Interestingly, Kallio et al. also reported the membranous and cytoplasmic locations of the EGFR immunostaining in RCC [25]. The different locations of EGFR immunostaining may be associated with progression and prognosis in RCC [26,27]. It is likely knowledge of the relationship between differential expression and cellular localization of EGFR and its ligands in normal and neoplastic lesions and patient survival might be beneficial in developing potential targeted agents for cancer therapy. Therefore, identifying the level and localization of EGFR expression in RCC is important for target-dependent therapy. However, characterization of distribution and localization of EGFR in normal kidneys and RCC tissues from the same patient have not been examined.

Thus we supposed the different locations of EGFR expression may be associated with human renal tumorigenesis. In this study, we examined the cellular localization of EGFR in RCC tumor portion and normal-looking renal cortical tissue from the same patient.

## Materials and methods

### Clinicopathological characteristics

This study had 63 patients with RCC, 46 males and 17 females with a mean age of 62 years. Each pair of tissues included a RCC tumor portion and normal-looking renal cortical tissue from the same patient. These specimens were obtained from nephrectomies carried out at the National Taiwan University Hospital (NTUH). Fuhrman's nuclear grading system from I to IV was used [28]. The grade I, II, III and IV classifications were present in 9 (14%), 29 (46%), 9 (14%), 14 (23%) cases and 2 cases (3%) were not determined, respectively. Tumors were staged according to the TNM system and histologically classified according to the WHO guidelines [29]. Tumors were further staged into 41 cases (65%) as being organ-confined (T1-2N0M0), 15 cases (24%) were locally advanced (T3-4N0M0) and 7 cases (11%) were metastatic (any T with N1-2 or M1). Clinicopathological characteristics of the tumors are summarized in Table 1. Approval from the Institutional Review Boards of NTUH and Kaohsiung Medical University were obtained and informed consent was received from all participating patients.

**Table 1:** Immunostaining expression of membranous EGFR in normal parenchymal and RCC tissues.

Characteristic	Patients	Membranous EGFR protein expression (mean score ± SE)		P value*
			
	No. (%)	Normal renal tubular cells	RCC	
Total				<0.001
Sex				
Male	46 (73)	0.1 ± 0.1	2.1 ± 0.1	<0.001
Female	17 (27)	0.2 ± 0.1	2.1 ± 0.2	<0.001
P value^†^		0.172	0.98	
Grade				
I	9 (14)	0.2 ± 0.1	1.9 ± 0.3	0.001
II	29 (46)	0.1 ± 0.1	2 ± 0.2	<0.001
III	9 (14)	0	2.3 ± 0.3	<0.001
IV	14 (23)	0.1 ± 0.1	2.2 ± 0.2	<0.001
ND	2 (3)	0	1.5 ± 0.5	
P value^‡^		0.679	0.641	
Stage				
Organ-confined (T1-2N0M0)	41 (65)	0.1 ± 0.1	2.1 ± 0.1	<0.001
Locally advanced (T3-4N0M0)	15 (24)	0.1 ± 0.1	1.9 ± 0.2	<0.001
Metastatic (N1-2 or M1)	7 (11)	0.1 ± 0.1	2 ± 0.4	0.002
P value^‡^		0.989	0.766	
Histological type				
Conventional	54 (86)	0.1 ± 0.1	2.2 ± 0.1	<0.001
Non-conventional	9 (14)	0.1 ± 0.1	1.3 ± 0.4	0.01
P value^†^		0.894	0.006	

NOTE: Expression of membranous EGFR was evaluated the intensity of membranous immunostaining and categorized as either 1+ (weak); 2+ (moderate) and 3+ (strong). Non-conventional type included papillary, chromophobe, sarcomatoid and collecting duct. Abbreviations: RCC, renal cell carcinoma; SE, standard error; TNM, tumor-node-metastasis; ND, not determined. *: paired sample t test, †: Independent-sample t test for two groups, ‡: One-way ANOVA for three or more groups.

### Immunohistochemistry (IHC)

Immunostaining was performed on paraffin sections by the avidin-biotin- peroxidase complex method, using a Super SensitiveTM Link-Label IHC Detection System (BioGenex, CA, USA). In brief, the sections were de-paraffinized in xylene and rehydrated through graded alcohols, then boiled in 0.01 M citrate buffer (pH 6.0) for 10 min. Hydrogen peroxide, 0.3%, was added to block any endogenous peroxidase activity. To block nonspecific binding the sections were incubated with a goat serum blocking solution composed of 10% normal goat serum in phosphate buffer saline, pH7.4 and 0.05% sodium azide. The sections were incubated with anti-EGFR antibody (Santa Cruz Biotechnology Inc, Santa Cruz, CA) used at 1:100 dilution at 4°C overnight. Horseradish peroxidase (HRP) polymer conjugated was used as a second antibody to avoid contaminating endogenous biotin or streptavidin (Zymed). After washing, the antigen-antibody complex was applied and stained with diaminobenzidine (Golden Bridge, Mukilteo, WA). Counterstaining was performed lightly with hematoxylin. Specific staining for EGFR was seen as a brown color in the cytoplasm or membrane, respectively. Breast cancer tissues served as a positive control of EGFR. Pre-immune serum was used instead of the first antibody as a negative control. All the control slides yielded negative results. Expression of EGFR was evaluated according to the ratio of positive cells and the staining intensity as described previously [30,31]. To semi-quantitate EGFR membranous and cytoplasmic staining, the following scoring method was applied. Expression of membranous EGFR evaluated the intensity of membranous immunostaining and categorized as either 1+ (weak); 2+ (moderate); 3+ (strong). Cytoplasmic staining was evaluated according to the ratio of positive cells and staining intensity. The ratio of positive cells each specimen was scored from 0~100% of the cells examined. Intensity was graded as follows: 0, no signal; 1, weak; 2, moderate and 3, strong staining. A total score of 0 to 300 was finally calculated (percentage of positive cells × staining intensity). The evaluation of immunostaining was performed by one pathologist (W. Y. K), was unaware of the tissue site and the fate of the patient.

### Isolation of membranous and cytoplasmic protein fractions

The cytoplasmic and membranous proteins were extracted from tissue samples using the ITSIPREP™ ProFEK KIT (ITIS Bioscience, PA, USA). About 50-100 mg of tissue was used and cut into small pieces. Ice-cold Cytosol Buffer 1 (0.08% potassium chloride, 0.02% magnesium chloride, 0.3% HEPES, 006% NP-40 and 0.005% EDTA) was immediately added using 5× the volume of the tissue, and homogenized with a homogenization device. The lysate incubated on ice for 15 min after being mixed with lysate by vortexing briefly and then centrifuging the sample at 3,000 × g for 5 min at 4°C. The supernatant was carefully collected and the pellet was retained. This pellet was the nuclear-protein enriched. Then the supernatant was centrifuged at 16,000 × g for 10 min at 4°C, and the supernatant was transferred into a clean storage tube. This fraction was the cytosol-protein enriched. Wash Buffer 2 (0.08% potassium chloride, 0.02% magnesium chloride, 0.3% HEPES and 0.005% EDTA) of was added to the nuclear pellet obtained above. The lysate was vortexed briefly and centrifuged at 3,000 × g for 5 min at 4°C. The supernatant was discarded and Nuclear Buffer 3 (4% sodium chloride, 0.02% magnesium chloride, 0.5% HEPES, 30% glycerol and 0.009% EDTA) was added. The lysate was incubated on ice for 30 min and vortexed every 10 min. And then the mixture was centrifuged for 10 min at 16,000 × g at 4°C. The supernatant was carefully collected and transferred to a clean storage tube. This fraction was the nuclear-protein enriched. The pellet was washed in the above step with Nuclear Buffer 3, vortexed briefly, and centrifuged as above. The supernatant was discarded and Total Membrane Buffer 4 (1% sodium chloride, 0.6% 1 M Tris-HCl pH 8.0, 1.3% NP-40, 0.6% deoxycholic acid, 0.6% sodium monohydrate and 0.2% sodium dodecyl sulfate) was added using 5× the volume of cell pellet. The lysate was incubated on ice for 30 min and vortexed every 10 min. It was then centrifuged at 16,000 × g or more for 10 min at 4°C. The supernatant was then carefully collected. This fraction was the membrane-protein enriched. The extracted membranous and cytoplasmic protein fractions were stored at -80°C for further analysis.

### Western blotting analysis

Cells scraped from one 100-mm Petri dish were resuspended in 100 μl of RIPA lysis buffer composed of 50 mM Tris-Cl, pH = 7.5, 1% NP-40, 150 mM NaCl, 10 mM EDTA, 1 mM sodium vanadate, 0.1% sodium dodecyl sulfate, 0.5% sodium deoxycholate, 10 μg/ml aprotinin, 1 mM phenylmethanesulfonyl fluoride, and 10 μg/ml leupeptin, and placed on ice for 30 min. The lysate was then centrifuged at 18,000 g for 30 min at 4°C to provide the supernatant for protein concentration determination. Cell extracts (50 μg) were separated on 10% SDS-polyacrylamide gels and transferred to immobilon polyvinylidene difluoride membranes (Millipore, Bedford, MA). After blocking, the membranes were incubated with human specific anti-EGFR (Santa Cruz Biotechnology) polyclonal antibody at 4°C for 12 h, followed by the horseradish peroxidase-labeled second antibody, and developed with the ECL system (Santa Cruz Biotechnology).

### Statistical analysis

Data are presented as the mean ± standard error of the means (SEM). Independent-sample t test and one-way ANOVA were used to compare protein expression determined by IHC analysis. Survival data was obtained from hospital and clinic records. The Kaplan-Meier method was used to estimate the probability of overall survival. The log-rank test was performed to examine the association of EGFR with overall survival. All tests were two-sided with *P *< 0.05 being statistically significant.

## Results

### Expression of membranous EGFR in normal parenchymal and RCC tissues

**Figure 1:**
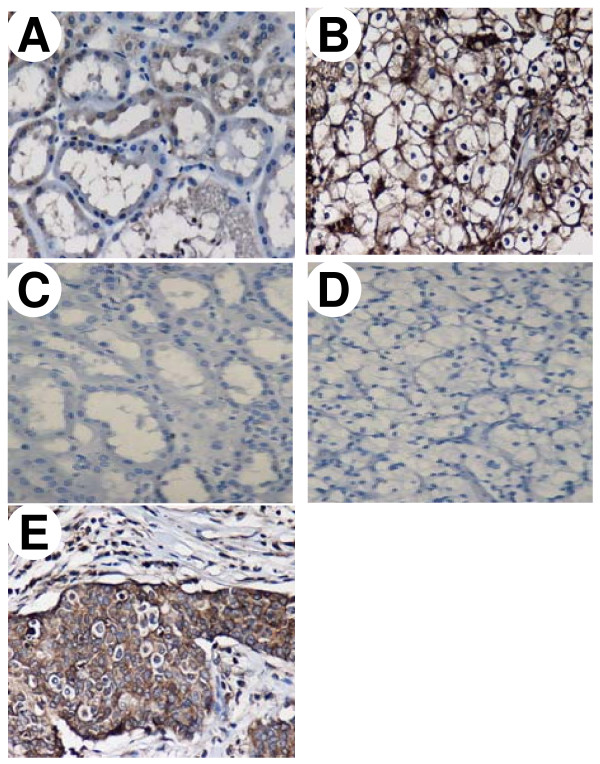
**Immunohistochemical staining of membranous EGFR in normal parenchymal and RCC tissues**. Tissue sections of normal parenchymal tissues (A, C) or RCC (B, D) were from the same patient. The polyclonal anti-EGFR antibody was used to stain paraffin sections (A, B). Negative control was the omission of the primary antibody (C, D). The breast cancer used as a positive control for EGFR expression (E). Original magnification, × 200.

**Figure 2:**
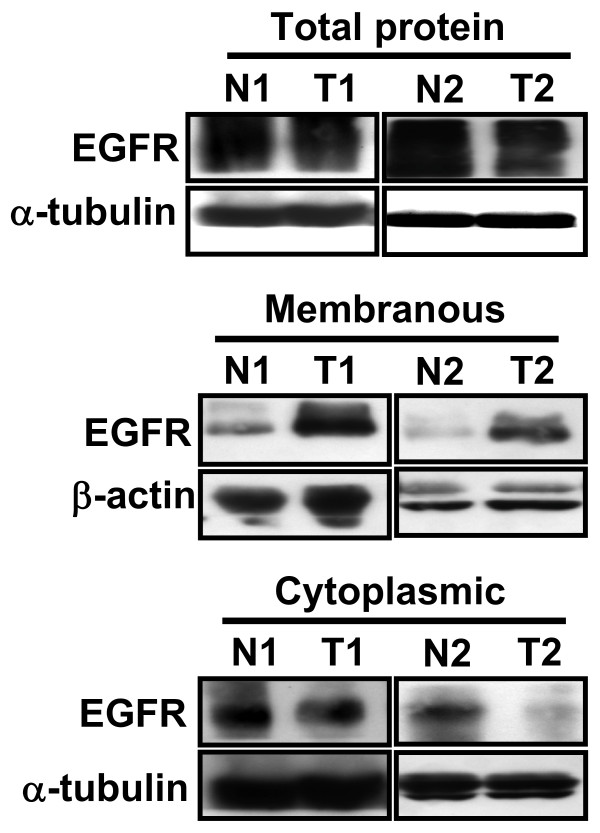
**Detection of membranous and cytoplasmic EGFR in normal parenchymal and RCC tissues**. The total, membranous and cytoplasmic protein fractions were extracted from tissue samples. The total (50 μg), membranous (80 μg) and cytoplasmic (50 μg) fractions were subjected to Western blotting with anti-EGFR antibody. β-actin and α-tubulin represented the loading internal controls, respectively.

The expression of EGFR protein was estimated in 63 pairs of tissues by immunohistochemistry (IHC). Membranous staining of EGFR was noted in normal parenchymal and RCC tissues. The EGFR protein staining was strongly positive in RCC cells but weakly positive in most normal parenchymal cells (Fig.1). IHC expression levels were further quantified on the intensity of membranous immunostaining and categorized as either 1+ (weak); 2+ (moderate); 3+ (strong). The IHC score was determined as the mean expression levels of normal and cancerous tissue cores analyzed respectively (Table 1). The RCC tissues had markedly elevated scores of 2.1 ± 0.1 compared to normal renal tissues scores of 0.1 ± 0.1 (*P *< 0.001) as presented in Table 1. The immunostaining of EGFR in normal tissues showed no signal stain in 88.9% (56 cases), weak stain in 9.5%% (6 cases), moderate stain in 1.6% (1 cases) and strong stain in 0% (0 cases) of the 63 pair specimens. In contrast, EGFR immunostaining in cancerous tissues showed no signal stain in 7.9% (5 cases), weak stain in 11.1% (7 cases), moderate stain in 47.7% (30 cases) and strong stain in 33.3% (21 cases) of these cases (data not shown). Similarly, the levels of membranous EGFR protein were increased in these RCC tissues as estimated by the protein fraction and Western blotting (Fig. 2). According to histological classification, membranous EGFR protein was significantly higher in conventional than in non-conventional RCC (*P *= 0.006). However, membranous EGFR levels in the RCC tissues did not differ between gender, age at diagnosis, nuclear grades and tumor stages (all *Ps *> 0.05), as shown in Table 1.

### Expression of cytoplasmic EGFR in RCC

**Figure 3:**
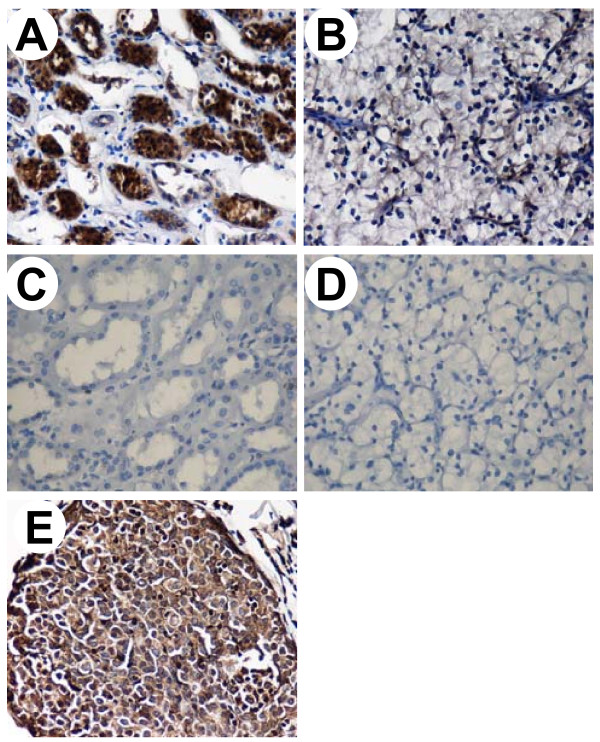
**Immunohistochemical staining of cytoplastic EGFR in normal parenchymal and RCC tissues**. Tissue sections of normal parenchymal tissues (A, C) or RCC (B, D) were from the same patient. The polyclonal anti-EGFR antibody was used to stain paraffin sections (A, B). Negative control was the omission of the primary antibody (C, D). The breast cancer used as a positive control for EGFR expression (E). Original magnification, × 200.

Specific staining of EGFR was noted in the cytoplasm. Thus, we examined the cytoplasmic staining of EGFR in normal parenchymal and RCC tissues by IHC. The IHC score was determined as the mean expression levels of normal and cancerous tissue cores analyzed, respectively (Table 2). The cytoplasmic EGFR protein staining was strongly positive in normal renal tubular cells but weakly positive in most cancerous epithelia (Fig. 3). IHC expression levels were further quantified on a scale from 0 to 300 (percent positive cells ± staining intensity, data not shown). The IHC score was determined as the mean expression levels of normal and cancerous tissue cores analyzed, respectively (Table 2). The normal renal tissues had markedly elevated scores of 201 ± 10.7 compared to RCC tissues scores of 103.3 ± 6.4 (*P *< 0.001) as shown in Table 2. Similarly, the levels of cytoplasmic EGFR protein were increased in these normal tissues as estimated by protein fraction and Western blotting (Fig. 2). However, the levels of cytoplasmic protein in the RCC tissues did not differ between gender, age at diagnosis, nuclear grades, tumor stages or histological types (all *Ps *> 0.05), as shown in Table 2.

**Table 2:** Immunostaining expression of cytoplasmic EGFR in normal parenchymal and RCC tissues.

Characteristic	Patients	Cytoplasmic EGFR protein expression (mean score ± SE)		P value*
			
	No. (%)	Normal renal tubular cells	RCC	
Total				<0.001
Sex				
Male	46 (73)	199.1 ± 13.1	106.7 ± 7.8	<0.001
Female	17 (27)	214.1 ± 18.5	92.9 ± 11.3	<0.001
P value^†^		0.54	0.345	
Grade				
I	9 (14)	176.7 ± 28.7	112.2 ± 17.9	0.048
II	29 (46)	209 ± 14.8	97.9 ± 9.9	<0.001
III	9 (14)	242.2 ± 20.1	122.2 ± 17.1	<0.001
IV	14 (23)	177.9 ± 28.2	96.4 ± 11.3	0.011
ND	2 (3)	240 ± 60	95 ± 65	
P value^‡^		0.257	0.556	
Stage				
Organ-confined (T1-2N0M0)	41 (65)	190.5 ± 13.4	94.9 ± 7.4	<0.001
Locally advanced (T3-4N0M0)	15 (24)	242 ± 18.5	120.7 ± 15.5	<0.001
Metastatic (N1-2 or M1)	7 (11)	194.3 ± 36.7	112.9 ± 18.1	0.099
P value^‡^		0.129	0.215	
Histological type				
Conventional	54 (86)	209.3 ± 10.8	102.8 ± 7	<0.001
Non-conventional	9 (14)	166.7 ± 38.2	104.4 ± 16.9	0.15
P value^†^		0.167	0.929	

NOTE: Expression of cytoplasmic EGFR protein was examined by immunohistochemistry on a scale from 0 to 300 (percent positive cells × staining intensity). Non-conventional type included papillary, chromophobe, sarcomatoid and collecting duct. Abbreviations: RCC, renal cell carcinoma; SE, standard error; TNM, tumor-node-metastasis; ND, not determined. *: paired sample t test, †: Independent-sample t test for two groups, ‡: One-way ANOVA for three or more groups

### EGFR expression and overall survival in RCC patients

**Figure 4:**
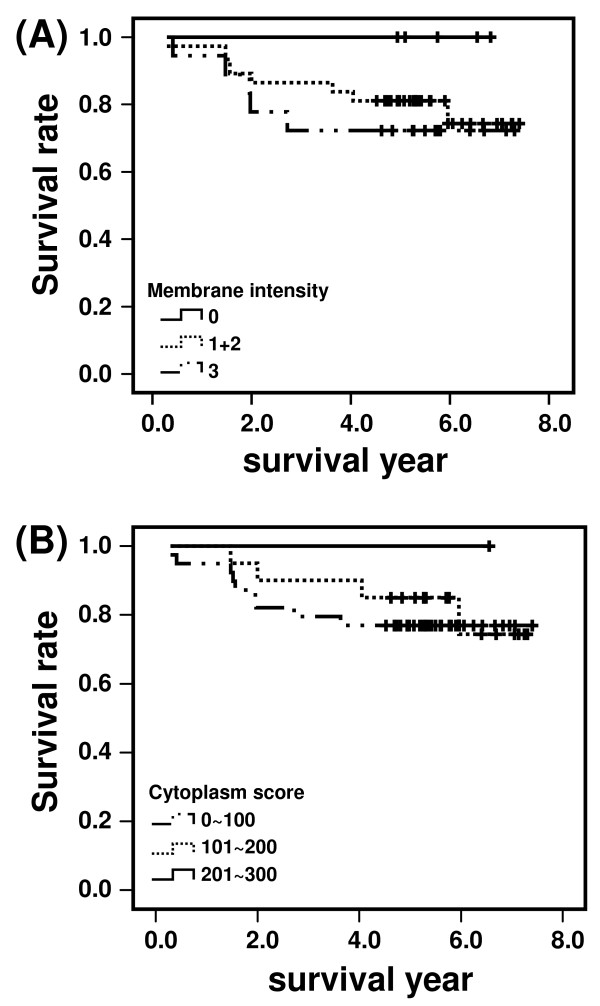
**Kaplan-Meier survival analysis of RCC patients**. (A) Correlation of membranous EGFR expression (negative, 0; positive, +1 to +3) with survival (years after surgery) in RCC patients. (B) Correlation of cytoplasmic EGFR expression (total score: 0~100, 101~200 and 201~300) with survival (years after surgery) in RCC patients. The log-rank test was performed to examine the association of EGFR with overall survival.

To examine if the different locations of EGFR expression correlated with survival of patients after surgery, Kaplan-Meier analysis was performed comparing EGFR-positive with EGFR-negative tumors in RCC patients. Our data showed a trend that RCC patients with positive expression of membranous EGFR had a poorer survival outcome compared with those with negative expression of membranous EGFR (Fig. 4A), although it did not reach statistical significance (*P *= 0.2). Similar trend as depicted in Figure 4B, showed lower expression of cytoplasmic EGFR in RCC patients had a poorer survival outcome compared with those with higher expression of cytoplasmic EGFR. Although it did not reach the significant correlation between expression of cytoplasmic EGFR and survival in RCC patients (*P *= 0.6). However, the small sample size may partially explain the lack of statistical significance.

## Discussion

In this study, we demonstrated the different locations of epidermal growth factor receptor (EGFR) immunostaining in renal tumorigenesis from renal cell carcinoma (RCC) and adjacent normal kidney tissues of 63 patients. EGFR expression in RCC was mostly located in the cell membrane, whereas the EGFR expression in normal renal tissues was chiefly seen in cytoplasm. Our results also were identical with previous studies showing higher expression of membranous EGFR frequently was detected and had a poorer survival outcome in many cancer cells. Here, our results suggested different locations of EGFR expression might be associated with human renal tumorigenesis.

Previous studies have shown EGFR overexpression in the advanced stage, poor prognosis and metastatic human cancer [32]. Over-expression of EGFR played an important role in tumor initiation and progression of RCC, so up-regulation of EGFR was correlated with high-grade tumors and a worse prognosis [33]. Advanced RCC was known to be largely resistant to conventional chemotherapy [34]. As a result, the prognosis for patients with advanced RCC was extremely poor [35]. Recently, both laboratory and clinical studies have shown the targeted agents for treatment of advanced RCC as a potential therapy. Therefore, this was particularly interesting because recently, anticancer therapies targeting the EGFR pathway have shown promising results in clinical trials of RCC patients [36,37]. The prognostic association of EGFR over-expression in RCC, however, is a controversial issue. Some studies showed an association of EGFR immunoreactivity with well differentiated RCCs [38], or regarded strong membranous EGFR immunostaining as an indicator of good prognosis [39], whereas others showed an association of EGFR immunoreactivity with high tumor stage/grade and poor prognosis [40], or no significant associations at all [41]. We found a similar result regarding the higher expression of membranous EGFR in RCC than in normal tissues. As expected, there was a trend that RCC patients with positive expression of membranous EGFR had a poorer survival outcome compared with those with negative expression of membranous EGFR. Further, our study showed there was a significant correlation between the level of membranous EGFR expression and histologic subtypes, with higher expression in conventional than in non-conventional RCC (including papillary, chromophobe, sarcomatoid and collecting duct). Previous studies indicated cytoplasmic EGFR immunostaining was associated with high tumor stage, grade and poor prognosis in RCCs [42,43]. The similar adverse prognostic [44] role of cytoplasmic EGFR has been shown in squamous cell carcinoma of the lung. Chandrika et al noted the different locations and level of EGFR expression in normal and cancerous lesions of lung [44], and suggested this switch from greater cytoplasmic EGFR to greater membranous EGFR expression might occur at the stage of dysplasia. Similarly, the aberrant cellular location of some adhesion molecules such as alpha-catenin may result in tumor dedifferentiation and aggressive, metastatic phenotype in laryngeal carcinoma [45]. Interestingly, we also found similar results that EGFR expression in RCC was mostly located in the cell membrane, whereas the EGFR expression in normal renal tissues was primarily occurred in the cytoplasm. Based on our results, we suggest different locations of EGFR expression may be associated with human renal tumorigenesis. However, the cellular localization (membrane *versus *cytoplasmic) of EGFR in RCC has not been addressed previously. The overexpression of EGFR in the cytoplasm of renal cortex may reflect receptor-ligand internalization, a rapid process occurring after ligand binding [46]. Although internalized receptors in most cell lines have been shown to be rapidly degraded [47,48], Dunn et al found a proportion of internalized receptor in EGF-treated hepatocytes was recycled to the cell surface [49]. However, we can't exclude the possibility intracellular localization of EGFR may be newly synthesized molecules within the endoplasmic reticulum or Golgi that have yet to be processed and inserted into the membranes.

Endocytic downregulation of signaling receptors has been regarded solely as a means of attenuating receptor signaling [50]. Recent study indicated that EGFR endocytosis was not only a way to inhibit activated receptors; it might also be a regulatory mechanism to control the expression of EGFR signaling [50,51]. Impaired endocytic downregulation of signaling receptors is frequently associated with cancer, since it can lead to increased and uncontrolled receptor signaling. In our study, may further demonstrate the mostly EGFR was expressed in cytoplasm through the regulatory mechanism of endocytic downregulation in normal kidney cells, whereas mostly EGFR can escape endocytic downregulation and expressed in membrane of RCC. However, further studies were needed to determine the biological role and clinical significance of EGFR location in RCC.

To our knowledge, this is the first study to demonstrate the EGFR location in normal and RCC tissues. Future studies should aim at answering the question of whether different patterns of immunoreactivity (membranous versus cytoplasmic) might help select patients for different approaches of EGFR targeted treatment.

## Competing interests

The authors declare that they have no competing interests.

## Authors' contributions

YS and CY planed the design of the study, participated in tissue collection and clinicopathological classification. YZ performed Immunohistochemical stain and Western blotting. WY participated in the evaluation of immunostaining score. GY, AM and SJ conceived of the study, performed the statistical analysis and assisted to draft the manuscript. HJ, MK, SP and WJ participated in tissue collection, clinicopathological classification and assisted to draft the manuscript. TC conducted the experiments, wrote the manuscript, and participated in its design and coordination. All authors read and approved the final manuscript.
